# Complete genomes of *Piscirickettsia salmonis* strains NZRLO1 and NZRLO2 isolated from salmon in New Zealand

**DOI:** 10.1128/mra.00339-25

**Published:** 2025-06-18

**Authors:** Ruy Jauregui, Timothy Hewison, Zac Waddington, Yee Syuen Low, Michelle McCulley

**Affiliations:** 1 Animal Health Laboratory, Biosecurity New Zealand, Ministry for Primary Industries 91821, Upper Hutt, New Zealand; 2 New Zealand King Salmon, Picton, New Zealand; University of Maryland School of Medicine, Baltimore, Maryland, USA

**Keywords:** fish pathogens

## Abstract

We report the complete genomes and plasmids of the Rickettsia-like bacterial isolates NZRLO1 and NZRLO2 from Chinook salmons (*Oncorhynchus tshawytscha*) in New Zealand.

## ANNOUNCEMENT

Rickettsia-like organisms (RLO) including *Piscirickettsia salmonis* (class: *Gammaproteobacteria*, order: *Thiotrichales*) and similar organisms are pathogens of relevance for the global fish farming industry (1). *P. salmonis*, identified as a pathogen in 1989, has since been found in Norway, Canada, Australia, and Chile, among others (2). Antibiotic treatment has delivered mixed results, and work has been ongoing for decades to optimize vaccines to protect farmed stock, for which information about genomic variability is of crucial importance (3, 4).

In New Zealand, isolates of NZRLO1 and NZRLO2 (5) were obtained from farmed New Zealand Chinook salmon (*Oncorhynchus tshawytscha*) from the Pelorus Sound (41.1628°S, 173.8632°E) in 2022 and the Queen Charlotte Sound (41.0815°S, 174.3332°E) in 2023 correspondingly. Skin and kidney tissue samples were tested on fish cell lines Epithelioma Papulosum Cyprini (EPC; ATCC:CRL-2872) and CHSE-214 (Chinook Salmon Embryo, ATCC:CRL-1681), and candidate isolates were characterized as either NZRLO1 or NZRLO2 via qPCR as described in (6). Isolates for each strain were re-grown on EPC cells, inoculates were grown in flasks at 15°C to log phase, and then subcultured onto specialized agar media, from which a single colony was selected for DNA extraction and whole-genome sequencing.

Genomic DNA was extracted using QIAamp DNA Kit (Qiagen, Hilden, Germany). Prior to library preparation for sequencing, genomic DNA was sheared using Covaris g-TUBEs (Covaris, Woburn, MA, USA) at 8,000 rpm for 1 minute, twice, to obtain DNA fragments of uniform length of 5 Kb. The fragment size, quality, and quantity of the DNA were analyzed using the Genomic DNA ScreenTape assay on the TapeStation 4200 system (Agilent Technologies, Santa Clara, CA, USA) and the Qubit 1× dsDNA High Sensitivity assay, respectively (Thermo Fisher Scientific, Waltham, MA, USA). Approximately 1–2 µg of DNA sample was prepared for Oxford Nanopore sequencing using the Ligation Sequencing Kit v14 (SQK-LSK-114XL; Oxford Nanopore Technologies, Oxford, UK). A PromethION R10.4.1 flow cell was primed, loaded with a prepared library, and sequenced on a PromethION 2 Solo device instrument (Oxford Nanopore Technologies, Oxford, UK). Base calling was performed using Dorado v7.0.2 with the high accuracy base-calling model v4.3.0. Dorado implemented a QC filter >Q9 and removed adapters as part of the base calling and demultiplexing process. Reads were assembled, circularized, and polished using Flye v2.9.5 (7).

Each assembly produced one circular, chromosome-sized fragment and smaller circular fragments identified as plasmids. Assembly completeness was evaluated using BUSCO and the thiotrichales_odb12 database (8), producing scores of 96.2% for NZRLO1 and 97.5% for NZRLO2. All programs used default parameters unless otherwise noted. Table 1 presents read numbers, genome statistics, and database links. Genomes were annotated by the automatic PGAP pipeline v6.9 (9) when uploaded to the NCBI GenBank database (NZRLO1: 3,441 genes, 6 rRNAs, and 54 tRNAs; NZRLO2: 3,444 genes, 6 rRNAs, and 56 tRNAs). These complete genomes are published in the hope of advancing our understanding of the relatedness of these endemic rickettsia-like strains to other published *Piscirickettsia* species. This will help in understanding virulence factors and possible routes for vaccine import and/or development.

**TABLE 1:** Sequence data and assemblies’ statistics

Raw reads				
Sample	**Read No.**	**Coverage**	**N50**	**SRA**
NZRLO1	2,591,644	2,500×	5.936 Kb	SRR31947490
NZRLO2	3,531,023	6,000×	7.285 Kb	SRR30992080
**Genome assemblies**				
Sample	**Fragment**	**Size**	**GC%**	**Genbank**
NZRLO1	Chromosome	3,076,404	39.4%	CP181382.1
	Plasmid 1	43,539	38.6%	CP181385.1
	Plasmid 2	131,761	38.4%	CP181383.1
	Plasmid 3	71,221	38.4%	CP181384.1
NZRLO2	Chromosome	3,043,193	39.6%	CP171655.2
	Plasmid 1	238,563	39.1%	CP178831.1
	Plasmid 2	44,977	37.8%	CP178832.1

## Data Availability

These Whole Genome Shotgun projects have been deposited in GenBank under the accession numbers PRJNA1208546 and PRJNA1172751. Individual sample links to GenBank and SRA are included in Table 1.

## References

[1] Chong RS. 2022. *Pisciricketsia salmonis* infection, p 433–438. In Kibenge FSB, Baldisserotto B, Chong RS (ed), Aquaculture pathophysiology, 1st ed. Academic Press, Cambridge, USA.

[2] Rozas M, Enríquez R. 2014. Piscirickettsiosis and Piscirickettsia salmonis in fish: a review. J Fish Dis 37:163–188. doi:10.1111/jfd.12211 24279295

[3] Maisey K, Montero R, Christodoulides M. 2017. Vaccines for Piscirickettsiosis (salmonid rickettsial septicaemia, SRS): the Chile perspective. Expert Rev Vaccines 16:215–228. doi:10.1080/14760584.2017.1244483 27690686

[4] Jaramillo D, Busby BP, Bestbier M, Bennett P, Waddington Z. 2024. New Zealand rickettsia-like organism and Tenacibaculum maritimum vaccine efficacy study. J Fish Dis 47:e13883. doi:10.1111/jfd.13883 37975241

[5] Brosnahan CL, Munday JS, Ha HJ, Preece M, Jones JB. 2019. New Zealand rickettsia-like organism (NZ-RLO) and Tenacibaculum maritimum: distribution and phylogeny in farmed Chinook salmon (Oncorhynchus tshawytscha). J Fish Dis 42:85–95. doi:10.1111/jfd.12909 30411368

[6] Gias E, Brosnahan CL, Orr D, Binney B, Ha HJ, Preece MA, Jones B. 2018. In vivo growth and genomic characterization of rickettsia-like organisms isolated from farmed Chinook salmon (Oncorhynchus tshawytscha) in New Zealand. J Fish Dis 41:1235–1245. doi:10.1111/jfd.12817 29806079

[7] Kolmogorov M, Yuan J, Lin Y, Pevzner PA. 2019. Assembly of long, error-prone reads using repeat graphs. Nat Biotechnol 37:540–546. doi:10.1038/s41587-019-0072-8 30936562

[8] Manni M, Berkeley MR, Seppey M, Simão FA, Zdobnov EM. 2021. BUSCO Update: novel and streamlined workflows along with broader and deeper phylogenetic coverage for scoring of eukaryotic, prokaryotic, and viral genomes. Mol Biol Evol 38:4647–4654. doi:10.1093/molbev/msab199 34320186 PMC8476166

[9] Li W, O’Neill KR, Haft DH, DiCuccio M, Chetvernin V, Badretdin A, Coulouris G, Chitsaz F, Derbyshire MK, Durkin AS, Gonzales NR, Gwadz M, Lanczycki CJ, Song JS, Thanki N, Wang J, Yamashita RA, Yang M, Zheng C, Marchler-Bauer A, Thibaud-Nissen F. 2021. RefSeq: expanding the prokaryotic genome annotation pipeline reach with protein family model curation. Nucleic Acids Res 49:D1020–D1028. doi:10.1093/nar/gkaa1105 33270901 PMC7779008

