# Correction for Jauregui et al., “Complete genomes of *Piscirickettsia salmonis* strains NZRLO1 and NZRLO2 isolated from salmon in New Zealand”

**DOI:** 10.1128/mra.00726-25

**Published:** 2025-08-07

**Authors:** Ruy Jauregui, Timothy Hewison, Zac Waddington, Yee Syuen Low, Michelle McCulley

## AUTHOR CORRECTION

Volume 14, no. 7, e00339-25, 2025, https://doi.org/10.1128/mra.00339-25. Page 2: An Acknowledgments section should be added which reads “The specialized agar media for the culture of Rickettsia-like organisms for this study were developed by Ryan Hunter and Karthiga Kumanan, Cawthron Institute, through the New Zealand Ministry of Business, Innovation and Employment Endeavour Programme-Emerging Aquatic Diseases: a novel diagnostic pipeline and management framework (CAWX2207).”

